# Difficulty Weaning From Cardiopulmonary Bypass Following an Aortic Valve Replacement

**DOI:** 10.7759/cureus.42692

**Published:** 2023-07-30

**Authors:** George Korelidis, Rory McFadyen, Chen Chuan Fang, Ghaith Qsous, Vipin Zamvar

**Affiliations:** 1 Cardiothoracic Surgery, Royal Infirmary of Edinburgh, Edinburgh, GBR

**Keywords:** aortic valve replacement (avr), cardiopulmonary bypass (cpb), coronary artery bypas grafting (cabg), tavr (transcatheter aortic valve replacement), intra-aortic baloon pump (iabp), surgical aortic valve replacement (savr), calcium debris, myocardial ischaemia

## Abstract

Aortic valve replacement (AVR) remains the treatment of choice for severe aortic stenosis. Despite the growing number of transcatheter AVR (TAVR) in younger and intermediate-to-low-risk patients, surgical AVR (SAVR) is widely used and retains low operative mortality, low rate of complications, and predictable long-term valve durability. Although it is a straightforward procedure, on some occasions, a surgeon could face challenging situations, such as compromised coronary flow and an inability to wean the patient from cardiopulmonary bypass (CPB). Our patient required concomitant coronary artery bypass grafting to overcome biventricular failure and facilitate successful weaning from CPB.

## Introduction

In this article, we present a case of a 78-year-old female patient who underwent aortic valve replacement (AVR) and was unable to be weaned off cardiopulmonary bypass (CPB) due to severe biventricular failure requiring coronary artery bypass grafting (CABG). Symptomatic severe aortic stenosis has a poor prognosis and should be treated either with surgical AVR (SAVR) or transcatheter AVR (TAVR). The European Association for Cardio-Thoracic Surgery (EACTS) guidelines 2021 make a class I recommendation (evidence level B) for SAVR over TAVR in patients with a low surgical risk (EuroSCORE II < 4%) and age < 75 years or in those who are operable and unsuitable for transfemoral TAVR [1].

## Case presentation

We present a 78-year-old female with a history of symptomatic severe aortic valve stenosis who was experiencing shortness of breath on exertion (New York Heart Association [NYHA] II). Her past medical history included non-ST elevation myocardial infarction (NSTEMI) in 2016 with percutaneous coronary intervention (PCI) to the left anterior descending coronary artery (LAD), hypercholesterolemia, gastroesophageal reflux disease, and cholecystectomy. The patient’s EuroSCORE II was calculated to be 1.15%. The preoperative echocardiography demonstrated severe aortic stenosis (Videos 1, 2).

**Video 1:** Severe aortic stenosis

**Video 2:** Severe aortic stenosis

**Figure 1:**
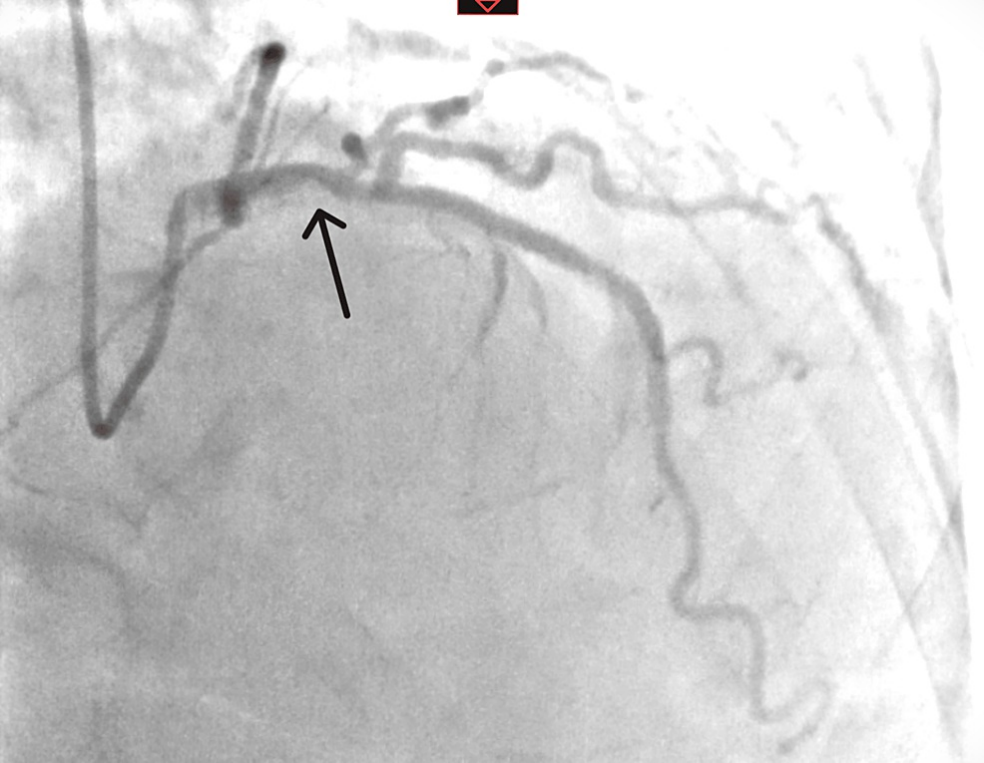
Nonsignificant proximal LAD stenosis reported <50% LAD: Left anterior descending.

The coronary angiography showed maximum 50% stenosis of the proximal LAD (Figure 1).

Operation

A standard midline sternotomy was performed, and CPB was instituted with cannulation of the distal ascending aorta and the right atrium. Cold blood cardioplegia was chosen, and the heart was successfully arrested shortly after its delivery. The native aortic valve had three cusps, all of which were heavily calcified, giving an impression of severe stenosis. The diseased native valve was excised, and a bovine bioprosthetic sutureless aortic valve (Perceval size L) was implanted.

The right coronary ostia was clearly visualized, and while the left coronary ostia was not clearly visible under a shelf of calcium, a good backflow was observed throughout the entire procedure.

After initially weaning from CPB, the trans-esophageal echocardiogram (TOE) showed good valve function, but the patient was placed back on full bypass due to hemodynamic instability and right ventricular failure. After remaining on CPB for a period of time, an attempt to wean from CPB failed again. A decision was made to deliver cardioplegia again and inspect the implanted valve. Concerns were raised about the possibility of compromised blood flow through the coronary ostia; therefore, it was decided to implant a smaller Perceval (size M) valve. However, despite good valve function, weaning from CPB failed again this time due to biventricular failure.

The decision was then made to perform CABG to both the right coronary artery (RCA) and LAD using vein grafts with proximal anastomoses to the ascending aorta. Following completion of the CABG, the patient was easily weaned from CPB without a need for an intra-aortic balloon pump (IABP), albeit with minimal inotropic support. TOE showed good biventricular contractility and an IABP was not required. The patient’s postoperative recovery was uncomplicated apart from an episode of atrial fibrillation, and she was discharged home on the sixth postoperative day.

## Discussion

CPB weaning difficulties following aortic valve surgery may be multifactorial with causes including compromise of coronary flow, cardiac arrhythmias, suboptimal myocardial protection, prosthetic valve regurgitation or severe para-valvular leak, electrolyte disturbance, and impaired thermoregulation [2]. Another potential reason for an inability to wean off from CPB is vasoplegic syndrome, which presents with low systemic vascular resistance, preserved contractility, and normal cardiac output [3].

In this instance, we hypothesize that the inability to wean successfully from CPB was due to coronary flow obstruction, given that grafting the RCA and LAD had an immediate positive effect, facilitating straightforward weaning from CPB. Coronary flow compromise could occur for various reasons such as preexisting or underestimated coronary artery disease, debris embolism from the displacement of a calcific plaque during native valve manipulation, excision or prosthetic valve deployment, left main artery dissection or injury during cardioplegia delivery [4], and inadequate de-airing of the heart [5].

Cardiac de-airing is a critical component in the weaning process; however, complete removal of the air from all cardiac chambers is not always possible, and air embolism can result in cerebral dysfunction, cardiac arrhythmia, or myocardial damage [6]. Therefore, standard techniques of cardiac de-airing are implemented as part of routine practice, such as CO_2_ insufflation, venting of the aortic root, shaking the heart, applying positive lung pressure to displace any air from pulmonary veins, and left ventricular apex aspiration. Although we did not use retrograde cardioplegia in this case, administering warm blood in a retrograde fashion for several minutes after removal of the aortic cross-clamp may reduce the risk of air embolism from any remaining air in the aortic root [7].

Compromising the flow through the coronary ostia due to the ring of a prosthetic valve or annular sutures is rare but accounts for a number of deaths after AVR [8]. Coronary ostial stenosis can also present months following the surgery, which may occur as a result of an injury during selective cardioplegia delivery or secondary to a fibrotic reaction on the aortic root from turbulent flow changes through the prosthetic valve. This can be treated either with CABG or PCI [6,9]. Coronary flow compromise following a TAVR procedure occurs in less than 1% but can significantly increase after TAVR in the TAVR procedure [10].

## Conclusions

In our case, the inability to wean from CPB was a consequence of compromised coronary blood flow, which could be explained either by underestimated preexisting coronary stenosis or displacing debris of calcium toward the coronary flow. It was unlikely to be compromised by a bioprosthetic Perceval valve as it was replaced to a smaller size and both coronary ostia were visible. Compromised coronary flow following AVR is a rare but potentially fatal complication. Surgeons must be aware of the multiple potential reasons for this complication, and CABG should be considered as a salvage procedure without delay to enable successful weaning from CPB.
